# Video assisted thorascopic assisted correction of left partial anomalous pulmonary venous connection: one case report

**DOI:** 10.1186/s13019-024-02501-8

**Published:** 2024-01-23

**Authors:** Gengxu He, Kai Zhou, Lei Zhao, Yuanzhi Luo, Hong Geng, Qiang Ji, Kun Zuo

**Affiliations:** Department of Thoracic and Cardiovascular Surgery, The First Affiliated Hospital of Hebei North University, Zhangjiakou City, Hebei Province P.R. China

**Keywords:** Partial anomalous pulmonary vein connection (PAPVC), Video assisted thoracoscopic surgery (VATS), Cardiopulmonary bypass (CPB), Computer tomography pulmonary angiography (CTPA)

## Abstract

**Introduction:**

The left partial anomalous pulmonary vein connection is a rare congenital heart disease, especially with intact atrial septum. Now we reported a case of the left superior pulmonary vein drainage to left innominate vein through a vertical vein, and corrected with video assisted thoracoscopy.

**Case presentation:**

A-59-years old man diagnosed left anomalous partial pulmonary vein connection with presentation of short breathiness and palpation, and diagnosed with computer tomography pulmonary angiography. The operation was carried out under video assisted thoracoscopy with one manipulation incision and one observational incision, the vertical vein was dissected and anastomosis with left atrial appendage. The patients recovered smoothly and postoperative CTPA showed anastomosis ostium was unobstructed.

**Conclusion:**

The left lateral thoracotomy and video assisted thoracoscopic surgery is a feasible for correction of left PAPVC with intact interatrial septum without using CPB.

## Introduction

Partial anomalous pulmonary vein connection (PAPVC) has a reported incidence between 0.4 and 0.7% [1]. The right side is more commonly affected, whereas the left side is affected up to 18,2% [2, 3]. The most common presentation is a right upper lobe vein draining into either the right atrium or superior vena cava. Only 3% of cases have been reported with drainage from the left lung into the innominate vein. PAPVC most commonly presents with an atrial septal defect (ASD), reportedly in 80–90% of cases. An intact atrial septum is extremely rare [4]. We reported this case with left partial anomalous vein connection was corrected through video assisted thoracoscopy without cardiopulmonary bypass.

## Case report

**Fig. 1:**
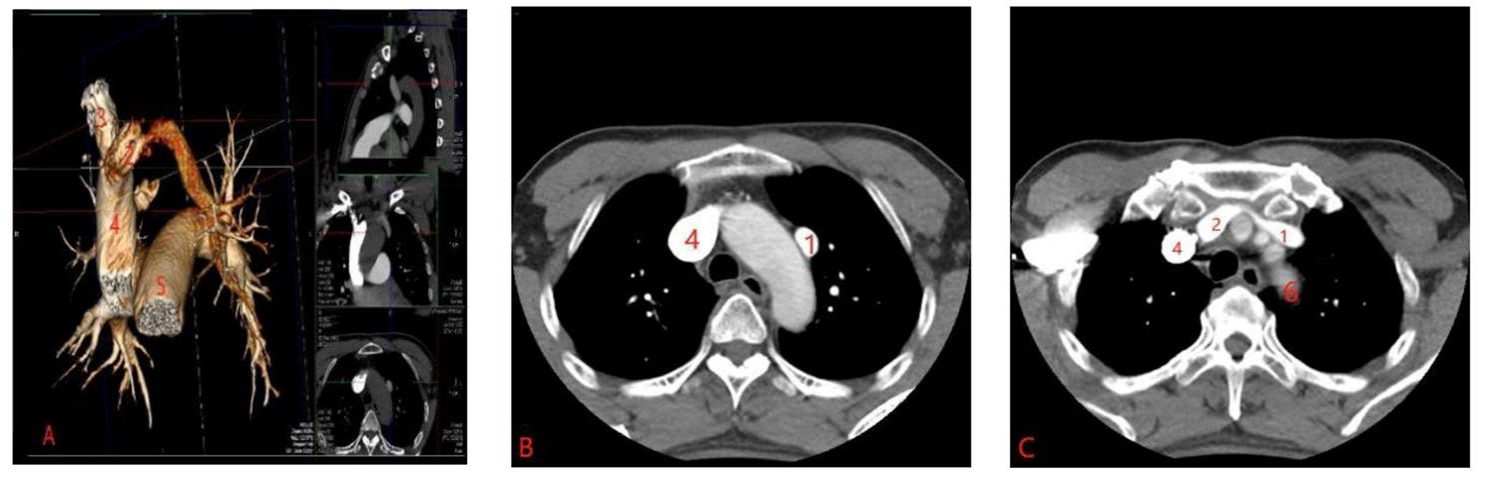
**A-C**: Preoperative CTPA and reconstruction of the pulmonary artery showed that the left superior pulmonary vein was connected to the left brachiocephalic vein and drained into the superior vena cava. (1. left vertical vein 2. left brachiocephalic vein. 3. right brachiocephalic vein. 4. superior vena cava. 5. pulmonary trunk. 6. descending aorta)

**Fig. 2:**
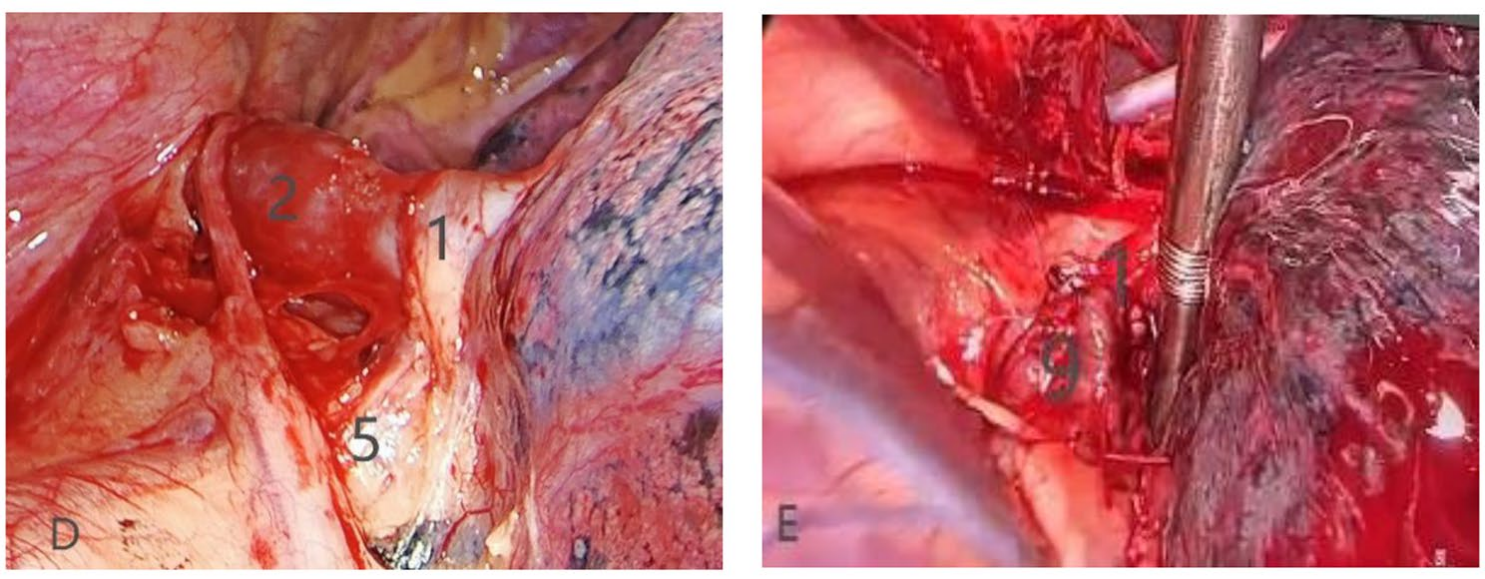
**D**: during the operation, the left superior pulmonary vein was connected to the left brachiocephalic vein; **E**: reconstruction of the connection between the left superior pulmonary vein and the left atrial appendage. (1. left superior pulmonary vein. 2. left vertical vein. 5. pulmonary trunk. 9. Left atrial appendage)

**Fig. 3:**
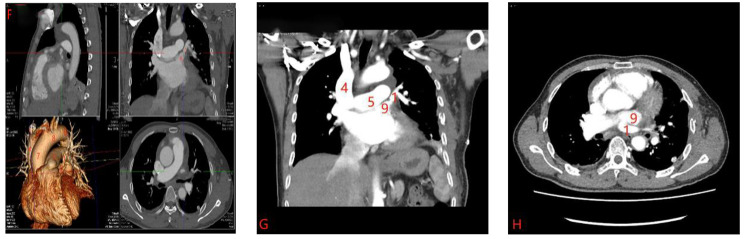
**F-H**: Postoperative CTPA after 7 days showed that the left superior pulmonary vein was well connected to the left atrial appendage. (1. left superior pulmonary vein. 2. left brachiocephalic vein. 3. right brachiocephalic vein. 4. superior vena cava. 5. Pulmonary trunk. 6. descending aorta. 7. ascending aorta. 8. aortic arch. 9. left atrial appendage)

A 59-year-old man who presented with short of breathiness and palpitation for 3 months was sent to our hospital, Physical examination revealed normal blood pressure and heart rate of 65 beats per minute. There was no jugular venous distention or left parasternal lift. A midsystolic 2+/6 murmur was heard over the precordium in the mitral area. The pulmonary and abdominal examinations showed no abnormalities. Transthoracic Echocardiography disclose the enlargement of right ventricle, and pulmonary artery systolic pressure of 51mmHg, with moderate tricuspid regurgitation, with intact atrial septum. Electrocardiography showed normal sinus rhythm, and left ventricle high voltage. We carried out the computer tomography pulmonary angiography (CTPA) of pulmonary artery and found that the dilation of pulmonary artery, and enlargement of right atrium and right ventricle, left superior pulmonary vein connected to the left brachiocephalic vein through a vertically orientated vessel courses lateral to the aortic arch, right pulmonary veins and left inferior pulmonary vein were seen draining normally into the left atrium (Figure 1A-C). The patient was diagnosed partial anomalous pulmonary venous connection. After careful examination, we performed the anastomosis of left superior vein and left atrial appendage through video assisted thoracoscopy without cardiopulmonary bypass. Two incisions were made, one was used as manipulation port about 5 cm length in the fourth intercostal space between the midaxillary line and midclavicular line, the other was 1.5 cm length in the seventh intercostal space at the midaxillary line. After exposure of the left pulmonary hilum, the vertical vein draining LSPV was dissected as long as possible to the confluence to the left brachiocephalic vein, and cut off at the confluence site using stapler, then the pericardium was opened behind the phrenic nerve and anterior left pulmonary hilum, the left atrial appendage was exposed, and the 50 mg heparin was administered into the vein by anesthetist, an auricular appendage clamp was placed on the left atrial appendage, and the vertical vein was clamped proximal to pulmonary hilum, a 20 mm opening was made in the vertical vein, another opening of similar dimension made over LA appendage. Vertical vein was anastomosed to atrial appendage with 5 − 0 prolene in side-to-side fashion (Figure 2D-E). After the anastomosis, the clamp at the pulmonary vein was removed and deair, the line was tied, and the auricle appendage clamp was removed. The upper part of pericardium was closed. And 24 F tube was inserted into left thoracic cavity. The postoperative recovery was uneventful. The CTPA was carried out 7th day postoperation, and it showed the left superior pulmonary vein connected to the left atrial appendage, the anastomosis was unobstructed (Figure 3F-H).

**Fig. 4:**
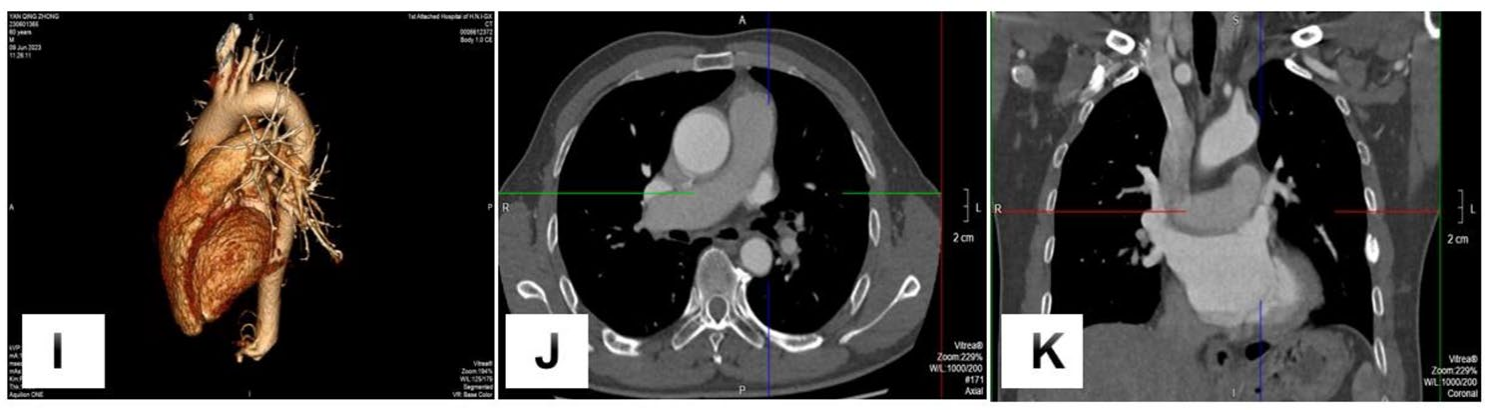
**I-K**: One year postoperative CTPA showed the left pulmonary vein connect to the left atrial appendage fluently

The patient was discharged after 7 days hospital stay postoperatively. CTPA was carried out every six months in the first year. CTPA after one year showed the left upper pulmonary vein connecting to the left atrial appendage fluently (Figure 4I-K).

## Discussion

The left PAPVC belongs to a broad spectrum of congenital anomalies resulting in a left to right shunt following failure of fusion of embryological pulmonary veins into the left atrium. The overall incidence is estimated between 0,4 and 0,7%. The right side is more commonly affected, whereas the left side is affected up to 18,2%.

The left superior pulmonary vein drainage into left brachiocephalic vein through a vertically orientated vessel is the commonest variation of the rarely left sided PAPVC, and even more so with intact interatrial septum. Left sided PAPVC forms a left to right shunt, which predisposes the patient to right ventricle volume overload, pulmonary artery hyperaemia, then pulmonary artery hypertension, right ventricular dysfunction and tricuspid regurgitation. The majority of the patients diagnosed with PAPVC presents with fatigue, dyspnea—both present in about 40% of the patients—and palpitations. Diagnosis can be difficult, missed, or only made at late clinical presentation in adulthood. As a useful method, echocardiography could evaluate the enlargement of right atrium and ventricle, and sometimes revealed the pulmonary veins opening into right atrium of left innominate vein [5]. CTPA is the most important technique to diagnose PAPVC, and clearly demonstrate the anatomy of this broad spectrum of pulmonary venous connection, and guide the surgical strategy [6]. This patient presented exertional dyspnea, and then diagnosed through CTPA.

Surgical correction has been reported in many reports, through the median sternotomy or lateral thoracotomy with or without cardiopulmonary bypass graft support [7–10]. We carried out the correction through a minor incision of left thoracotomy under video assisted thoracoscopic. The left orientated vertical vein could be easily dissected and cut off at the confluence site, the length of the vein is enough to extend to anastomosis with left atrial appendage. And the morphology of left atrium will affect the anastomosis, a larger left atrial appendage will make the anastomosis easier. For the left atrial tissue is relatively fragile, no tension anastomosis is crucial during the operation. If the length of the vertical vein is not enough or the left atrial appendage is small, it is essential to use an external tunnel to be interposed between the left upper pulmonary vein and the left atrium to reduce the tension, just as reported by Hioki. Previous surgical case reports have shown that the correction of left sided-PAPVC does carry the risk of complications such as atrial fibrillation, complete heart block, cardiac arrest, and pulmonary venous obstruction [9, 10], but such complications were not encountered in our this case, we believe that no tension anastomosis and proper size of anastomosis orifice are important to avoid bleeding and right superior pulmonary vein obstruction.

We believed that the left lateral thoracotomy and video assisted thoracoscopic surgery is a feasible for correction of left PAPVC with intact interatrial septum.

## Data Availability

The datasets used and/or analysed during the current study are available from the corresponding author on reasonable request.
